# Editorial: New insights on the transmission and pathogenicity of rickettsiae

**DOI:** 10.3389/fcimb.2023.1183558

**Published:** 2023-03-27

**Authors:** Hua Niu, Xiaolu Xiong

**Affiliations:** ^1^ Laboratory of Hepatobiliary and Pancreatic Surgery, Affiliated Hospital of Guilin Medical University, Guilin, China; ^2^ Guangxi Key Laboratory of Molecular Medicine in Liver Injury and Repair, Guilin Medical University, Guilin, China; ^3^ Guangxi Health Commission Key Laboratory of Basic Research in Sphingolipid Metabolism Related Diseases, Affiliated Hospital of Guilin Medical University, Guilin, China; ^4^ State Key Laboratory of Pathogen and Biosecurity, Beijing Institute of Microbiology and Epidemiology, Beijing, China

**Keywords:** rickettsiae, vector transmission, natural reservoir, effector protein, virulence mechanism

The rickettsiae are a diverse collection of obligate intracellular Gram-negative bacteria infecting ticks, lice, fleas, mites, chiggers, or mammals. Many members of rickettsiae cause human diseases through vector-borne or airborne transmission (Azad and Beard, 1998). However, the knowledge on their prevalence in endemic areas and transmission between hosts is still limited (Karim et al., 2021). Moreover, due to their obligate intracellular lifestyle, rickettsiae have developed numerous strategies to subvert host cellular processes and evade immune surveillance to promote their replication, leading to disease development (Voss and Rahman, 2021). Research on their pathogenicity will broaden our knowledge on pathogenic mechanisms of rickettsiosis. With this Research Topic, we have collected five research articles, one review article and one opinion article, which described the recent advances made in these fields.

Spotted fever group Rickettsia (SFGR) are a group of pathogens that mainly maintained and transmitted by ticks. In China, more than 10 species of SFGR have been detected, but the information regarding the prevalence of SFGR in ticks is still limited. Qi et al. determined the prevalence of SFGR in ticks from wild hedgehogs and domestic bovine in Jiangsu province, China and evaluated the threats posed by these SFGR to public health. Their results provide useful epidemiological data for prevention and control of potential rickettsial infections.

In contrast to other SFGR pathogens, *Rickettsia felis* can be carried by different vectors such as fleas, ticks, mites, lice, and mosquitoes. The first human case of *R. felis* infection in China was reported in 2014, and reported cases increased in recent years. In a retrospective survey, Teng et al. identified four cases of *R. felis* infection in China between 2021 and 2022 by real-time PCR and semi-nested PCR, highlighting the risk posed by this pathogen to public health in China.

Using meta-transcriptomic sequencing, Zhang et al. investigated the microbiome and virome of skin biopsy specimens from tick-bite patients and analyzed pathogens including rickettsiae potentially transmitted from skin to blood. This work reported human skin infectome after a tick-bite from patients, illustrating the real risk of ticks posing on human health and highlighting more attention should be paid on the cutaneous response to prevent tick-borne illness.

A review article by Huang et al. summarized the rickettsial effectors involved in subverting diverse host cellular processes, including membrane dynamics, actin cytoskeleton dynamics, phosphoinositide metabolism, intracellular trafficking, and immune defense. This review article provides new insights on how these virulence factors attenuate the host’s innate immune defense and orchestrate the permissive replication niche.

As the causative agent of Q fever, *Coxiella burnetii* promotes its colonization inside of host cells by transferring effector proteins into host cytoplasm through type IV secretion system (Fu et al., 2022b). To date, the function of most of the effector proteins remains elusive (Fu et al., 2022a). A recent study demonstrated that *C. burnetii* utilizes an effector protein adapted from glycolysis to subvert host immunity (Zhang et al., 2022). An opinion article by Guo and Sun further discussed the importance of this finding to the current knowledge of mechanisms utilized by intracellular bacteria to evade host immunity.

*Ehrlichia chaffeensis*, the causative agent of human monocytic ehrlichiosis (HME), infects and multiplies in human monocytes and macrophages. Upon infection, host immune cells produce reactive oxygen species (ROS) to kill pathogens, but the mechanisms exploited by *E. chaffeensis* to cope with ROS remain largely unknown. Using ChIP-seq, biochemistry assay and PNA transfection, Liang et al. demonstrated that *E. chaffeensis* CtrA activates the expression of glutathione S-transferase, which confers oxidative stress resistance to the bacterium. This work not only uncovers the mechanisms exploited by *E. chaffeensis* to utilize GSH to combat ROS damage, but also provides useful information for the development of new therapeutics against HME.

*E. chaffeensis* resides and replicates in a cytoplasmic vacuole, named as *Ehrlichia*-containing vacuole (ECV). Revealing the molecular composition of ECV is important in understanding the host cellular processes, evasion of host defense pathways and in defining host-pathogen interactions. Kondethimmanahalli and Ganta analyzed the proteomes of ECV and identified many novel ECV membrane-associated bacterial proteins such as p28-Omps. This study represents the first comprehensive investigation of ECV membrane proteome and demonstrates the extensive modification of ECV membrane by the pathogen. The results are valuable to define pathogenic mechanisms critical for the replication of the pathogen within macrophages.

Collectively, this Research Topic not only provides updated knowledge into current understanding on the transmission and pathogenicity of rickettsiae, but also provides new ideas for control and prevention of rickettsial diseases. Recent advances in technology such as next generation of sequencing and CRISPR-based gene editing will facilitate further research on these obligate intracellular bacteria.

## Author contributions

All authors listed have made a substantial, direct, and intellectual contribution to the work and approved it for publication.
